# The Localized Discovery and Recovery for Query Packet Losses in Wireless Sensor Networks with Distributed Detector Clusters

**DOI:** 10.3390/s130607472

**Published:** 2013-06-07

**Authors:** Rui Teng, Kenji Leibnitz, Ryu Miura

**Affiliations:** 1 Wireless Network Research Institute, National Institute of Information and Communications Technology, Yokosuka 239-0847, Japan; E-Mail: ryu@nict.go.jp; 2 Center for Information and Neural Networks, National Institute of Information and Communications Technology, and Osaka University, Osaka 565-0871, Japan; E-Mail: leibnitz@nict.go.jp

**Keywords:** sensor networks, query loss, immune systems, query loss discovery, collective recovery

## Abstract

An essential application of wireless sensor networks is to successfully respond to user queries. Query packet losses occur in the query dissemination due to wireless communication problems such as interference, multipath fading, packet collisions, etc. The losses of query messages at sensor nodes result in the failure of sensor nodes reporting the requested data. Hence, the reliable and successful dissemination of query messages to sensor nodes is a non-trivial problem. The target of this paper is to enable highly successful query delivery to sensor nodes by localized and energy-efficient discovery, and recovery of query losses. We adopt local and collective cooperation among sensor nodes to increase the success rate of distributed discoveries and recoveries. To enable the scalability in the operations of discoveries and recoveries, we employ a distributed name resolution mechanism at each sensor node to allow sensor nodes to self-detect the correlated queries and query losses, and then efficiently locally respond to the query losses. We prove that the collective discovery of query losses has a high impact on the success of query dissemination and reveal that scalability can be achieved by using the proposed approach. We further study the novel features of the cooperation and competition in the collective recovery at PHY and MAC layers, and show that the appropriate number of detectors can achieve optimal successful recovery rate. We evaluate the proposed approach with both mathematical analyses and computer simulations. The proposed approach enables a high rate of successful delivery of query messages and it results in short route lengths to recover from query losses. The proposed approach is scalable and operates in a fully distributed manner.

## 1. Introduction

With sensing, computation, and wireless communication capabilities, wireless sensor networks (WSN) enable distributed sensing and processing of natural phenomena applied to areas such as environmental or healthcare monitoring. In a sensor network, a sink node requests data from the sensor nodes by sending queries to them. A query is typically a data-centric operation [1,2] instead of conventional address-centric operations as used in current Internet protocols. For example, a query might be “*what is the temperature in the geographical region X?*” or “*what is the humidity in region Y?*” rather than “*what is the humidity level at node A?*”. Nodes with data matching to the query interest will report this information back to the sink node via a multi-hop transmission.

However, a query packet may not successfully reach the corresponding sensor nodes that should reply to the query, either due to packet loss in the wireless propagation or sensor nodes being temporarily unavailable. This in turn results in the failure of sensor nodes to report their information correctly to the sink. Since the query does not identify an individual destination node, query loss would lead to the losses of data sources, which may cause a wrong evaluation of the situation in the monitoring region with respect to the query.

Many studies on sensor networks assume that the query dissemination process is reliable and focus their discussions on the data collection [3–5]. This can be a reasonable assumption when the data collection accounts for much more traffic volume than query dissemination or the failures of queries have only little impact on the success of data collection. However, when the queries have a large impact on the network traffic, for example, in case of on-demand queries of sensing data from various end users, retransmission of queries will cause large traffic overhead, and recovering from query losses has an essential impact on the network traffic and energy consumption.

The broadcast feature of query message dissemination is a reason that leads to both problems of query losses and the difficulty of their local recovery. Due to the large number of sensor nodes in the network, the broadcast packet collisions cause the loss of query messages. Since a broadcast process does not adopt the confirmation by ACK in the packet delivery in order to avoid ACK storms, it is difficult to enable a local retransmission of query messages at the sensor nodes that lost query messages.

There are a number of studies on the improvement of broadcast efficiency with regard to broadcast overhead, energy efficiency, and reliability. These efforts include designing specific structure based broadcast mechanisms including utilizing cluster, tree, or backbone based structures for broadcast [6–9]. Reducing the redundancy of broadcast has the effect of reducing broadcast collisions. However, these specific structure-based broadcast methods can not completely guarantee to be without loss of broadcast packets, which are caused not only on MAC layer but also on physical layer at small sensor nodes.

A further development that does not depend on the network infrastructure is with local queries using NACK at each sensor node [10], leading to distributed and localized discovery of query loss. However, such approaches require the setup of a query sequence. In case the sequence of queries is over a long interval, the discovery and recovery must wait until the new query with a new sequence to judge whether there is a lost sequence number among its received query packets.

A straightforward approach is the utilization of the sink node for query loss discovery and recovery since the sink node is the source of the query and also the destination of data collection from the sensors that should respond to that query. To enable sink nodes to detect query losses, they should have the knowledge of which sensor nodes should reply to a query. A sink that can detect a query loss at a sensor node recovers a query loss by resending the same query to that sensor node. In large networks, this recovery of query loss requires a much longer route than a local recovery of query losses near the point of failure.

Our objective is to enable localized and distributed query loss discovery and recovery in query dissemination. The desired operation of localized discoveries and recoveries from query losses includes not only the capability of localizing the scope of discoveries and recoveries, but also the capability of automatically focusing on the query losses particularly at the nodes that are corresponding to the query. This target is essential for large size sensor networks and the sensor networks that have various user queries or support the diversity of sinks. The distributed operation here specially requires the desired approach to be spatially and temporally independent of sinks, since widely applied sensor networks should support diversity of sinks rather than only one sink node with fixed position. The reason for localized operation of query discovery and recovery is to enable the scalability of queries in large sensor networks.

To achieve the above goals, we design a localized discovery and recovery approach that requires only local cooperation at sensor nodes to cope with query losses. The proposed approach is scalable in large sensor networks and enables a high success rate of loss discovery and recovery. In the proposed approach we particularly employ the following techniques to achieve the design goals: (1) distributed loss detector clustering for sensor nodes so as to enable localized discovery and recovery of query losses, where each sensor node can be a loss detector of its neighbor node; (2) distributed name resolution at each detector to enable localized and selective response to a query; (3) collective node operations in discovery and recovery to improve the success rate.

Both mathematical analyses and computer simulations are conducted to verify the effectiveness of the proposed approach. We give mathematical analyses to examine the properties of the proposed approach including collective impact of loss discovery and recovery, as well as scalability. Furthermore, we examine the features of cooperation and competition at MAC and PHY layers in the processes of loss recoveries. We conduct computer simulations to verify the performance improvements with regard to the success of query delivery, scalability, and the impact of detector numbers.

The rest of the paper is organized as follows. Section 2 surveys the related work. Section 3 describes the problems of query losses and recoveries in sensor networks. Sections 4 and 5 describe and analyze the proposed approach of the localized query loss discovery. Section 6 presents the simulation results of the proposed schemes and Section 7 concludes the paper.

## 2. Related Work

In [10], a reliable query approach was proposed with the goal of having an efficient discovery and recovery of query packets. The approach lets a sensor node that discovers a query packet loss send out a NACK message to the sink. The sink recovers this loss by delivering the query message to the node again. To detect the loss of a query packet, sensor nodes adopt the NACK mechanism based on utilizing a query sequence at the sink node. The success of this approach depends on the condition of continuous queries and the approach is difficult to be used for on-demand queries from various users. The delays of query discovery and recovery may be large when the interval between two continuous queries is large.

Directed diffusion discussed about the basic operation of data queries in sensor networks [5]. A naming mechanism for query and sensing data is presented. The proposed approach finds reliable routes for data delivery based on the route's confidence degrees. In that work, query broadcast is assumed to be ideal, *i.e.*, each sensor node always successfully receives every query message from the sink node.

There are approaches that have been proposed to enable the sensor-to-sink reliability in sensor networks, such as Event-to-Sink Reliable Transport (ESRP) [11] and Pump Slowly Fetch Quickly (PSFQ) [12]. However, most of these approaches focus only on the reliable data delivery in sensor networks from sensor to sink and the broadcast of data queries is generally assumed to be successfully performed or not discussed.

Many approaches of local route recovery have been proposed also in application to mobile ad hoc networks [13,14]. A basic idea of local route recovery is utilizing alternative relay nodes to build a new connection to the destination. However, these approaches are especially proposed for end-to-end unicast routing, which does not require specific detection processes for a route failure, since a node can know that it encounters a route failure when it cannot deliver a packet to the next hop node in its routing table.

In [7], a reliable tree-based broadcast is proposed. The target is to reduce the significant overhead of packet broadcast in sensor networks. Their basic idea is to construct a spanning tree among sensor nodes in the network rooted at the source of broadcast. Their proposed approach reduces redundant packet transmissions. By neighbor requirement and packet ordering, reliable broadcast is realized for tree-based broadcast. The local repair of node failure is a parent-child based interaction. The paper does not discuss the cost of tree formation from various roots of sinks, which will lead to a large overhead for constructing the tree.

Active query forwarding has an aim of energy efficient and localized query dissemination and then reduces the redundancy in flooding based query [15]. The basic idea is that it allows each node that carries the query message to get knowledge of the topology of its *k*-hop neighbors. Therefore, each node enables a trajectory forwarding of query messages to the related nodes. The proposed approach ACQUIRE highly reduces the flooding redundancy. On the other hand, the requirement of *k*-hop neighbor information leads to a large traffic of information updating, especially when sensor nodes that correspond to a query have been sparsely deployed.

## 3. Problem Statement

**Figure 1.**
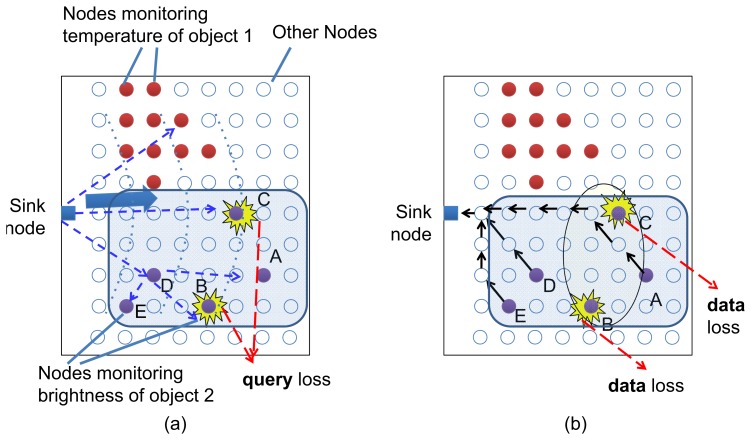
**(a)** Data query and **(b)** data collection in WSN. Loss of data query messages leads to loss of sensing data.

We consider a sensor network that operates in a data-centric way in which the queries do not need to identify an individual destination node. As shown in Figure 1, in order to collect sensing data, the sink node disseminates a query that describes the data of interest, e.g., temperature or humidity, to the network. Upon receiving a query message that matches their data, the corresponding sensors reply to this query. The sink node is not concerned with knowing which node should reply or whether sensor nodes have received the query. This will cause an essential transport problem that the sink node will not detect sensing source loss in the network. For example, in Figure 1 the nodes *A*, *B*, *C*, *D*, and *E* should reply to a query of brightness data, but nodes *B* and *C* do not receive the query due to loss or node failure. In this case, the sink node will not know that sensor nodes *B* and *C* are also data sources for this query. In the case that *B* and *C* have critical and unique data that are essential to this query, this situation will lead to an incorrect judgment and decision at the sink node.

Due to the large number of queries from various users and the large size of sensor networks, it is difficult to adopt conventional reliable broadcast schemes with high complexity. Furthermore, packet losses caused by temporary failures of sensor nodes cannot be solved by any reliable broadcast method.

Detection and recovery of packet losses in the query dissemination at the object nodes are essential to enable the success of data collection. In case the sink node has all information of which nodes should reply to each query, a general approach can be that the sink node recovers the loss of query by retrieving the data again from those nodes. Since unicast can be more reliable and easily controlled, the recovery is generally reliable with regard to the success of packet delivery. This approach is straightforward and can be widely applied assuming that each sink node knows every sensor node that is corresponding to each query in the entire network.

However, this sink based recovery approach suffers from the problem of scalability with increasing sensor network size. The route length of recovery refers to the length of the route between the sink node and an object sensor node. Such route length increases with the network size. In case there are several query losses that need to be recovered, the sink node will have a large cost for recovering over a large distance. The large length of recovery increases the network traffic and recovery delay and consumes much energy at sensor nodes. Therefore, a scalable detection and recovery mechanism of query losses is essential for sensor networks, especially for those with large network size.

## 4. Localized Query Loss Discovery with Distributed Query Resolution

**Figure 2.**
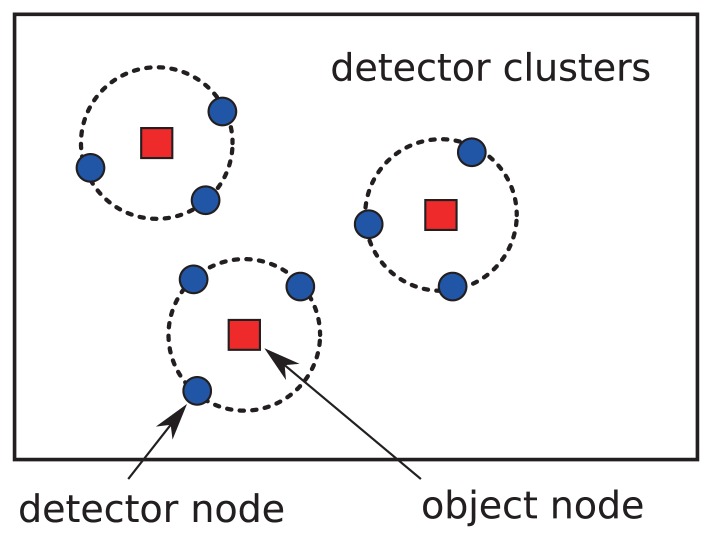
Distributed detector clusters in sensor networks.

We propose a localized query loss discovery to efficiently detect query loss problems in sensor networks. Before introducing the details of the proposed approach, we at first describe the basic idea of the proposed approach. Each sensor node in the network is provided with collective loss detectors that are composed of a cluster of local neighbor nodes. The cluster is termed *detector cluster* and the sensor node being monitored in the cluster center is termed the *object node*. As shown in Figure 2, each sensor node is an object node, around which clusters of detectors are assigned to collectively detect the query loss at the object sensor node.

By employing a distributed query resolution mechanism at detector nodes, a detector is able to have a localized and selective response to each query that are correlated to the objects that the detector is monitoring. When a detector finds that a query is correlated with one of the objects that it is monitoring, it starts to monitor the query loss by utilizing wireless overhearing of data reply messages from the object. A detector will judge that there is a query loss at the object when there is no data reply overheard, since the object that is correlated to a query should reply sensing data to the query. Note there are multiple detectors for an object to detect the query losses. The success rate of loss discovery is expected to highly increase since there are collective discoveries of query losses by the multiple detectors that have the spatial-temporal diversity in regard to wireless communication.

The ideas in the proposed approach take some insights from the immune system (IS), which adopts the teamwork of immune cells to efficiently detect and eliminate pathogens [16–18]. The natural immune system is a complex adaptive system that protects the body (organism) from invading pathogens such as viruses or bacteria, *etc.* An immune system consists of two major procedures: *pathogen detection* and *pathogen elimination*. All these are performed in a distributed and localized manner. Pathogen detection operates by distinguishing “self” and “non-self” cells in the immune system. When a pathogen is detected, the corresponding immune cells are activated to eliminate the pathogen. Pathogen elimination in the adaptive immune response copes with the problem of choosing the right receptors for the particular kind of pathogen to be eliminated [16]. For example, the activated B-cell will produce antibodies that have a Y-shaped structure, and can collectively bind to the pathogens.

### 4.1. Detector Cluster Formation for Localized Query Loss Detection

The cluster formation algorithm is shown in Algorithm 1.



**Algorithm 1** Detector Cluster Formation (DCF) Algorithm
**Require:** Node ID, attributes, the scope (*T*) of random start time**Ensure:** Detector cluster1:*t* = current time2:*T*_0_ = *t*3:Randomly select a cluster broadcast time *T_start_* = *T*_0_ + *Rand*(0 − *T*)4:**if**
*t* = *T_start_*
**then**5: Perform 1-hop cluster invitation broadcast with (node ID, location, attributes)6: Set number of cluster invitation broadcasts = 17: Wait {a randomly selected duration, e.g., 10 times of the duration for a round of invitation packet delivery and reply}8:**end if**9:**while** (the number of replied nodes < threshold reply) **and** (the number of cluster invitation broadcasts < threshold invitation) **do**10: Receive the neighbor response message of “join as a detector”, which is sent by unicast from neighbor node with (node ID, location, attributes) information.11: Increase the number of cluster invitation broadcasts12: Wait13:**end while**14:Perform 1-hop confirmation broadcast with (node ID, cluster size)


An object node invites its one-hop neighbor node to perform the role of loss detector by broadcasting an invitation message. The broadcast is initiated at a random time to prevent collisions of clustering operations at other object nodes. The nodes that receive the invitation message send a reply of “join as a detector” to accept being the object node's detector.

The invitation message includes node ID, location, and sensing attributes of the object sensor nodes. This message lets neighbor detectors have the information for correlating between a query and the object node. If there are not enough nodes to perform the role of detectors, the object node will initiate the invitation again until there are sufficient detectors or the number of query broadcasts is large enough.

A cluster of detectors around an object node recognizes the loss of a query at the object node that should respond to it. The query loss is detected based on the judgment of the following two processes:

1. A detector detects that the query is associated with the object node that it is monitoring. This is realized by a distributed query resolution mechanism at each detector node.
2. The object node does not respond to the query. This information is captured by the detector by receiving or overhearing the wireless transmission of the object node.

When the above two processes occur, a query loss is detected at a detector.

**Proposition 1.** *Sensor nodes can achieve functional fairness with regard to being loss detector and object*.

*Proof*. Let a sensor node A have a set of detectors *D* = {*d_1_*, *d_2_*, …, *d_n_*}. Since node A is the neighbor node of each detector *d_1_*, …, *d_n_*, when node A serves as detector for *d_1_*, *d_2_*, …, *d_n_*, the function of being detector and object are mutually shared for *A* and all nodes in *D*. In the same way, every sensor node can be a detector of its object and then the network has the functional equality among nodes.

We summarize the basic procedure of cluster formation as follows. (1) Sensor node *A* sends an invitation to its neighbors by broadcast; (2) Neighbor nodes reply with “join as detector” by unicast; (3) The node *A* sends an ACK to the detectors by broadcast.

### 4.2. Distributed Query-Address Resolution (QAR)

To efficiently detect and recover the query losses, each detector should judge whether a query is associated with the object node that the detector is monitoring. If an object node is associated with the query, the detector is activated to monitor the query losses at the object. On the other hand, if the detector finds that a query is not associated with the objects it is in charge of, the detector can go to inactive mode (such as sleep mode) to save energy.

The basic function of *Query-Address Resolution* (QAR) is in some way analogous to that of judgment of “pathogen harmfulness” in immune systems. It enables each detector node in sensor networks to make a correct decision of whether to “remove” a query loss by initiating a query loss discovery. QAR resolves the query at each individual sensor node to the corresponding addresses of sensor nodes and it lets each loss detector judge whether a query is correlated with the object node of a detector. Each node registers its ID, location and attributes, such as temperature or humidity, to its detectors. A query message can be resolved to the corresponding sensor addresses by the QAR.

**Figure 3.**
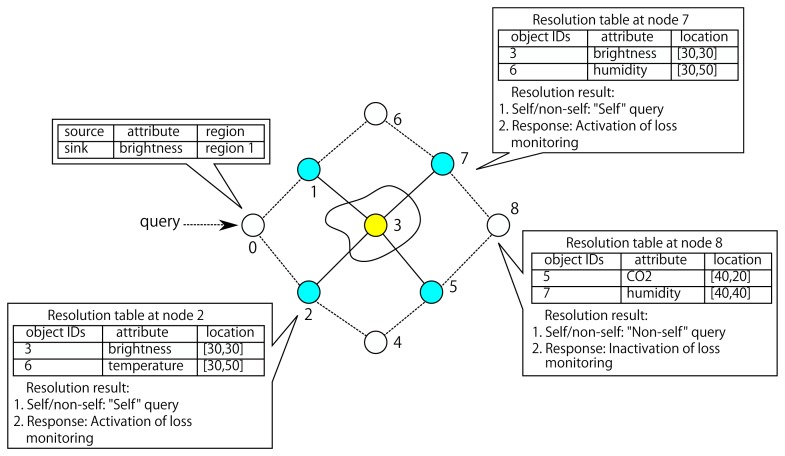
Query to address resolution table at loss detectors.

An example of the information maintained at QAR of a node is illustrated in Figure 3, where Node 3 registers its ID, location and attributes to its loss detectors Nodes 2 and 7. Upon receiving a query message “*what is the brightness in the region 1?*”, the detectors are able to judge that node with ID 3 in the resolution table should give a response. The detectors will initiate a loss recovery if they detect that the node with ID 3, rather than other nodes, has not replied to the query.

**Figure 4.**
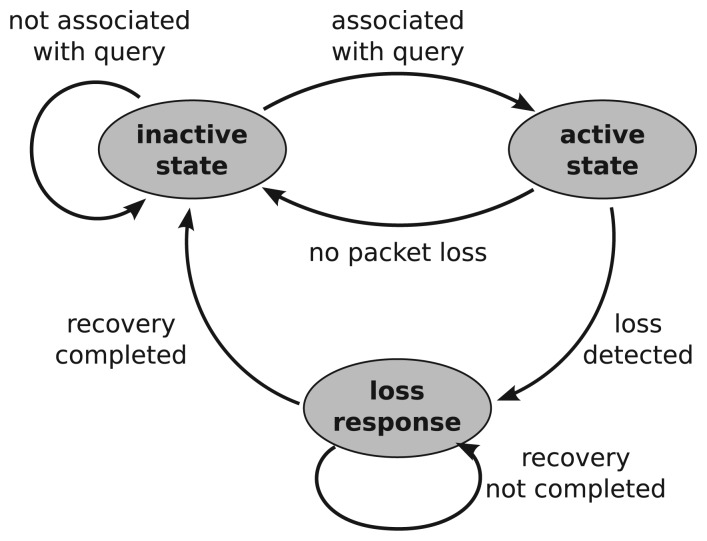
State transition of a detector node.

The query-address resolution enables each detector node to have a selective and localized response to each query, which is correlated with a certain type of sensor nodes rather than all sensor nodes [1,2,5]. That is, each detector adopts an activation/response manner to start working only when a query loss is found to be correlated with its object sensor nodes. Figure 4 shows the state transitions at a detector node. The detector node is in its activated state when it receives a query message requesting data from one of the objects that it is monitoring. The detector node then decides whether it should respond by checking the query loss of its monitored objects. This is realized by overhearing packet replies from the object nodes. If there is packet loss, the detector initiates the recovery process in which each detector of the object node sends a recovery packet in turn to recover from packet loss at the object node. When the detector node receives a confirmation of recovery or the time of the recovery process expires, the detector returns to its initially inactive state.

### 4.3. Collective Query Loss Detection

Let *P* denote the probability of a query loss at an object sensor node in the query dissemination. Then, we know that the successful query delivery probability at a sensor node is *S*_1_ = 1−*P*. For *n* detectors of an object node, the successful detection of query loss requires at least one of the detectors to successfully receive the query packet. Since the probability that all nodes lose the query packet is *P^n^*, the probability of successful query delivery to at least one of the detectors is *S*_2_= 1 − *P^n^*. Suppose that a detector that successfully received a query packet can successfully detect a query loss of its object node. Then, *S*_1_ is the probability of query loss detection by a single detector for its object node to lose the query packet. Furthermore, *S*_2_ is the probability of query loss detection by a cluster of detectors for its object node that loses the query packet. Hence, the ratio of successful detection of query loss at an object node between the single detector approach and the collective detector based approach is shown in Equation (1).

(1) $$R_{\text{success}}=\frac{S_{2}}{S_{1}}=\frac{1-P^{n}}{1-P}$$

**Figure 5.**
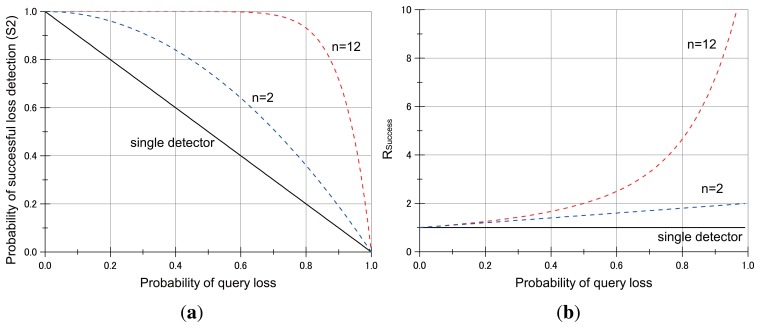
**(a)** The probability of successful query loss detection for a detector cluster (*S*_2_) and **(b)** *R_success_*.

Figure 5 illustrates the impact of utilizing loss detectors on the successful detection of query losses by varying the probability of a query loss. The utilization of collective detectors keeps a successful delivery probability that is at least equal to or higher than that of single query detector. For example, in case that the number of loss detectors of a sensor node is *n* = 12, the success probability of the collective detector approach is maintained above 90 % when the loss probability *p* < 0.8, and the ratio is up to 4.5 times to that without utilizing the collective loss detectors.

### 4.4. Scalability of Loss Discovery

The scope of loss discovery in the rebroadcast-based approach is the whole network with *N* nodes. On the other hand, the proposed approach requires only *n* one-hop neighbor nodes for query loss discovery and recovery. Therefore, the ratio of query loss discovery in the conventional approach to the one with local detectors is as shown in Equation (2).

(1) $$R_{\text{scope}}=\frac{n}{N}$$

Equation (2) can be interpreted as follows. For every query loss in the standard flooding method, all *N* nodes need to be involved in the rebroadcast process. On the other hand, when using local detectors, the scope of rebroadcasts is limited to only the *n* neighboring nodes at the node where the query was lost. Since we usually have N ≫ n, our approach can significantly limit the traffic overhead caused by rebroadcast messages and it scales linearly with the number of neighbors per node.

## 5. Localized Recovery of Query Losses

### 5.1. Localized Query Loss Recovery

Loss elimination is a local process to recover from query loss at a node. Compared with the conventional centralized approach, the local recovery has very low overhead and is able to perform a fast recovery. The basic concept is similar to utilizing a growing number of immune cells and the recovery procedure is a dynamic collaborative operation. It increases recovery efficiency and avoids recovery collisions among detectors with a growing reaction to the query loss.

**Figure 6.**
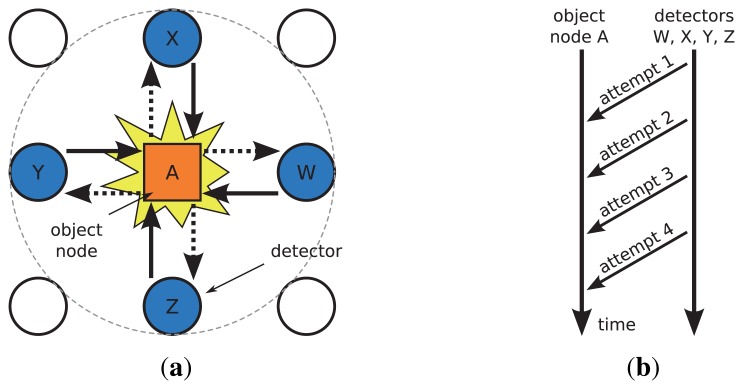
**(a)** A detector cluster in loss recovery; **(b)** Collective recovery of query packet loss.

As shown in Figure 6(a), the proposed approach adopts a recovery operation with multiple detectors such as *W*, *X*, *Y*, and *Z*. The query packet that has not arrived at node *A* is cached at each detector. When a query loss is recognized at node *A*, the detectors (nodes *W*, *X*, *Y*, *Z*) attempt to deliver recovery messages that include the query content. Each detector contends for the access to the wireless media in a random manner, which is widely used in wireless communication and leads to a simple and easy setup of the wireless network. If one recovery attempt from a detector fails, another detector will attempt to deliver the recovery message to the node *A* again, see Figure 6(b). Due to the wireless broadcast, if node *A* responds to the recovery packet by delivering sensing data to the sink, the detectors *W*, *X*, *Y*, and *Z* will overhear this sensor data packet. If a detector node receives the response from node *A*, it will stop sending the recovery message to *A*.

### 5.2. The Route Length of Recovery (RLR)

In the conventional approaches of localized recovery of query loss, the sink node plays a central role to recover from failure of receiving query packets at a sensor node. In [10], the retransmission of query packets to a sensor node that lost the query message can be localized by using unicast from sink to a sensor node, assuming that every query is disseminated to all sensor nodes in the network and each sensor node knows the route to the sink.

We compare the sink based recovery approach with the proposed approach by analyzing the *route length of recovery* (RLR), which is defined as the minimum route length (in hops) that is taken in the recovery operation. RLR reflects the effectiveness of localization in the query loss recovery. In the calculation of route length, we assume that the link between any two nodes in the sensor network is bidirectional.

As shown in Equation (3), in the proposed recovery scheme, the RLR *L_IS_* consists of one hop for loss notification and retransmission of the recovery packet. While in the conventional sink-based approach, the RLR *L_SB_*, as shown in Equation (4), includes the number of hops *R*(*sink*, *i*) from sink to a sensor node *i* for loss notification and the same number of hops from the sink to the sensor for the retransmission of the recovery packet.

(3) $$L_{\text{IS}}=1$$

(4) $$L_{\text{SB}}=2R(\text{sink},i)$$

**Figure 7.**
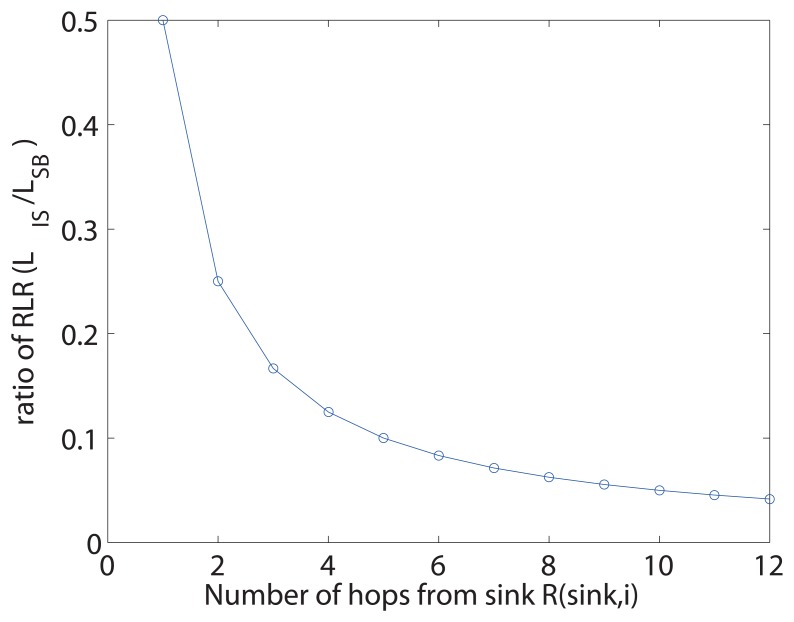
Ratio of the route length of recovery (RLR) between the proposed method and sink-based approach.

Figure 7 illustrates the ratio of RLR between *L_IS_* and *L_SB_* for varying route length from sink to the sensor where query loss occurs. The RLR of sink-based recovery grows linearly with the distance between the sink to where the loss occurs, while our proposed method is independent of the location of the loss, which gives an exponential decrease in ratio.

### 5.3. The Impact of Detector Number on Successful Recovery

We study the impact of the number of detectors on the performance of packet loss recovery. The target is to highlight the correlation between detector number and the effectiveness of immune inspired operation.

Let *n* be the number of detectors that perform recovery of an object at which the query packet is lost. We analyze the packet loss recovery at an object node *A*. The recovery is performed after a query loss was detected at the object *A*. The detectors of object *A* recover the loss by unicasting the recovery packet to object *A*. The recovery unicast from a detector faces contention with other detectors at MAC layer as well as the network layer packet loss.

Suppose the MAC protocol adopts random media access with node contention. Although there are various MAC protocols, we adopt random access due to its high channel utilization capability [19,20] and its simplicity for small sensor nodes. Random access is the commonly utilized contention based MAC scheme in technologies such as WLAN, Zigbee, *etc*. If the recovery contention window size is *W*, the detector recovers the query loss detected in a random time within (0, *W*).

Let *P_RS_* be the probability of recovery success for a detector to send a recovery packet to its object node. Note that the probability of recovery success of a detector is under the condition that

1. MAC layer success: there is no collision with other detectors at the time of recovery packet delivery,
2. PHY layer success: successful PHY layer unicast of the recovery packet to the object.

Let *P_PHY_* be the probability of recovery unicast failure at PHY layer to deliver a packet from the neighbor detector nodes. Further, let *P_MAC_* be the probability of recovery failure at MAC layer to deliver a packet from neighbor detector nodes. Therefore, we have

(5) $$P_{\text{RS}}=(1-P_{\text{PHY}})\;(1-P_{\text{MAC}})$$

Note a MAC success for a detector indicates that there is no collision with any of the other *n* − 1 detector nodes in a contention window *W*. Suppose each MAC access time is one time unit in a contention window of *W* time units. By regarding the media access operation as Bernoulli trials without sequence correlation, we can calculate *P_MAC_* as

(6) $$P_{\text{MAC}}=1-{\left(\frac{W-1}{W}\right)}^{n-1}$$

Then, we can rewrite Equation (5) as

(7) $$P_{\text{RS}}=(1-P_{\text{PHY}}){\left(\frac{W-1}{W}\right)}^{n-1}$$

Let *P_RF_* = 1 − *P_RS_* be the probability of recovery failure for a detector to send a recovery packet to its object node. Then the probability that all detectors of an object fail to recover can be written as

(8) $$P_{\text{RF}}^{*}={\left(P_{\text{RF}}\right)}^{n}$$

leading to the overall probability of successful recovery

(9) $$\begin{array}{cc} P_{\text{succ}} & =1-P_{\text{RF}}^{*}\\ & =1-{\left(1-(1-P_{\text{PHY}}){\left(\frac{W-1}{W}\right)}^{n-1}\right)}^{n} \end{array}$$

To illustrate the impact of the detector number on PHY layer performance in recovery, we allow *W* to be large enough so that the probability of success in MAC is ${\left(\frac{W-1}{W}\right)}^{n-1}\approx 1$. Then we can approximate Equation (9) as

(10) $$P_{\text{succ},\text{PHY}}=1-P_{\text{PHY}}^{n}$$

To illustrate the impact of the number of detectors on MAC layer performance in recovery, we allow *P_PHY_* to be 0. Then we can rewrite Equation (10) as

(11) $$P_{\text{succ},\text{MAC}}=1-{\left(1-{\left(\frac{W-1}{W}\right)}^{n-1}\right)}^{n}$$

We now briefly discuss some examples. Let *W* = 10, and *P_PHY_* = 0.5. At first we can plot the impact of the detector number on PHY layer performance in recovery according to Equation (10), where

(12) $$P_{\text{succ}}=1-P_{\text{PHY}}^{n}=1-0.5^{n}$$

**Figure 8.**
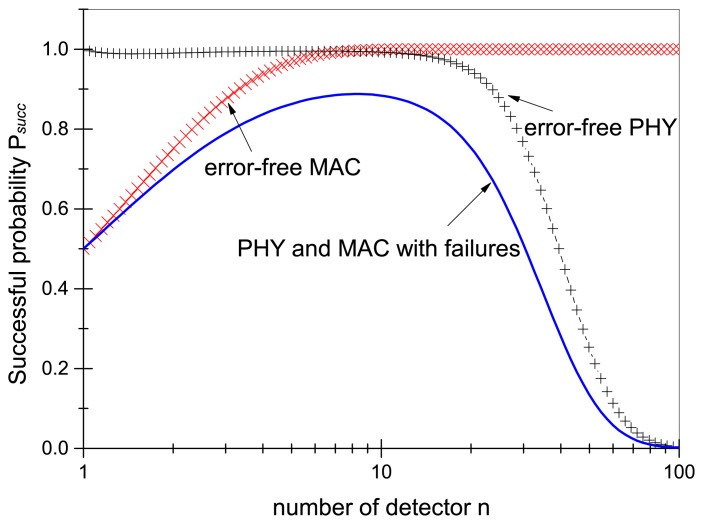
Delivery success probability *P_succ_* with ideal MAC and PHY, as well as integrated model, for varying number of detector nodes *n*.

The result in Figure 8 shows that the larger the number of detectors is, the higher the probability of successful recovery in the PHY layer performance with error-free MAC becomes. This is because the more detectors there are, the more diversity in recovery unicast exists, leading to a larger probability of overcoming the PHY layer packet losses due to the fading, multipath propagation, *etc.* The second curve in Figure 8 shows the impact of detector number on MAC layer performance in recovery according to Equation (11) with error-free PHY. The larger the number of detectors is, the lower is the probability of successful recovery in the MAC layer performance. This is because more detectors cause more contentions in recovery, leading to a higher probability of recovery packet collisions. The third curve shows the integrated recovery result of both PHY and MAC layers according to Equation (9) with errors on both MAC and PHY layers. There is an optimal detector number that achieves the highest probability of successful recovery. Too few detectors result in poor performance on PHY diversity, while too many detectors result in poor performance on MAC layer.

## 6. Simulation Evaluation

We also evaluate the proposed approach with computer simulation. We use C++ based simulations to study the proposed protocol of localized discovery and recovery of query losses. We focused our simulation study mainly on the network layer performance. We also adopted a simplified random access MAC with various contention windows.

The evaluations examine the performance of the proposed approach validating the impact of the proposed approach on the successful query delivery, scalability of loss discovery, *etc.* In our simulations, we compare the proposed protocol with the conventional query dissemination, which is a query broadcast operation among sensor nodes and is widely applied in the information retrieval in sensor networks, such as directed diffusion, knowledge based sensor networks, *etc.* [2,5]. The major purpose of our comparison is to validate the effect of the proposed approach on the improvement of successful query dissemination and its feasibility for sensor networks that have a large number of sensor nodes.

To compare the query recovery operation, we also adopt sink-based recovery in the simulation, since it is a straightforward potential method in sensor networks and has been proposed by researchers [10]. In sink-based recovery, the sink node checks the received sensor data and retrieves the lost data from those nodes that have not successfully delivered their data to the sink. The sink-based approach requires the sink node to maintain the IDs of all sensors that should reply to each query.

We evaluate the proposed and conventional approaches with the following metrics.

1. *The number of object nodes that have query packet losses*: The average total number of object nodes that suffer packet losses in a query among the sensor nodes that should reply to the query. This metric reflects the quality of the query dissemination process and has an impact on the success of sensor data collection from object nodes.
2. *The average route length in the recovery of query losses*: The average hop length of a route from the object node that fails to receive a query message to the detector node or sink node that recovers the query loss.
3. *The number of successful recoveries*: The total number of successful recoveries in the simulation with varying number of detectors.

In the simulation, we try to examine the impact of the network size, the number of object nodes that should respond to a query, and the number of detectors per sensor node. We particularly study the scalability of the proposed approach and the related approaches. The scalability in performance is essential to sensor networks that have large network size and resource constraints.

Unless otherwise stated, the simulations are set up as follows. The number of sensor nodes varies from 10 to 100. Each sensor is equipped with a radio module that has a transmission range of 10 m. The sensor nodes are distributed over rectangular areas: 100m × 10m for 10 nodes, 100 m × 20 m for 20 nodes, 100 m × 30m for 30 nodes, and 100m × 100 m for 100 nodes. The default number of detector nodes for each object node is 4. As for the broadcast in the simulation, each node randomly selects a start time to broadcast a received message within a time window of 0–5 time units. The probability of packet loss in recovery is set to 20 %. In the route selection, shortest path routing is utilized in the simulation.

**Figure 9.**
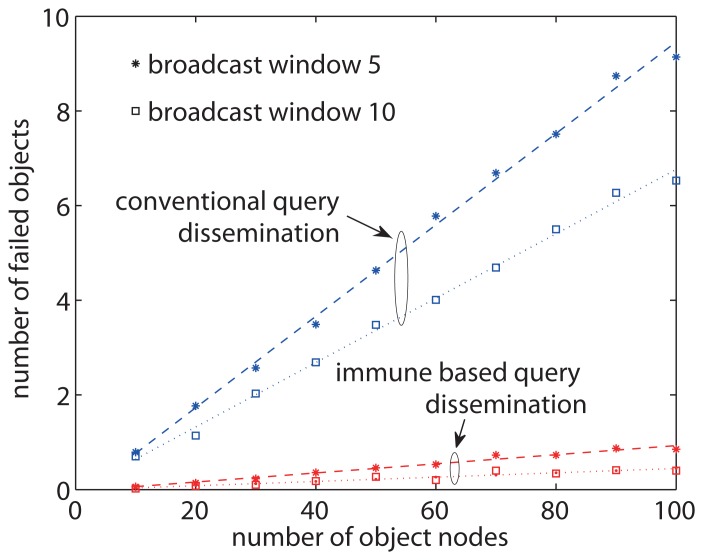
The average number of object nodes that have query losses with broadcast windows of 5 and 10 time units. The plots illustrate the scalability performance of successful query with regard to the number of object nodes.

Figure 9 shows the average number of object nodes that failed to receive a query message in query dissemination in the sensor network with 100 nodes. As for using conventional query dissemination in general sensor networks, the sink node may not have all sensor information in the network, hence we do not assume that the sink node performs loss recovery here. The number of objects that lose the query messages increases significantly as the total number of object nodes increases. The largest number of lost object nodes is up to 9 and 6 depending on the broadcast window size. On the other hand, the proposed approach allows only few query losses at object nodes and maintains a low number of failed object nodes as the total number of object nodes increases, leading to good scalability in network performance. When the total number of object nodes increases to 100, the number of failed objects in the proposed approach is only about 11 % and 17 % to that of the conventional approach. This is because the proposed approach utilizes local detection and recovery, and is only little affected by the network size. A small time window improves the broadcast speed and decreases the delay and energy consumption, but may cause more message losses at object nodes.

In the evaluation of the proposed approach and related conventional approaches, the sink's position affects the performance in the recovery of query losses. We set up two types of sink node positions: defined position and random position. As for the defined position of the sink node, there are two kinds of setup corresponding to two positions, which are in the top-left corner and on the middle top edge. As for the random sink position, the position of the sink node is selected randomly in the network area in each query. The operation of the proposed approach utilizes the local recovery until all detectors fail the recovery. A sink based recovery will be initiated if all detectors fail the recovery.

**Figure 10.**
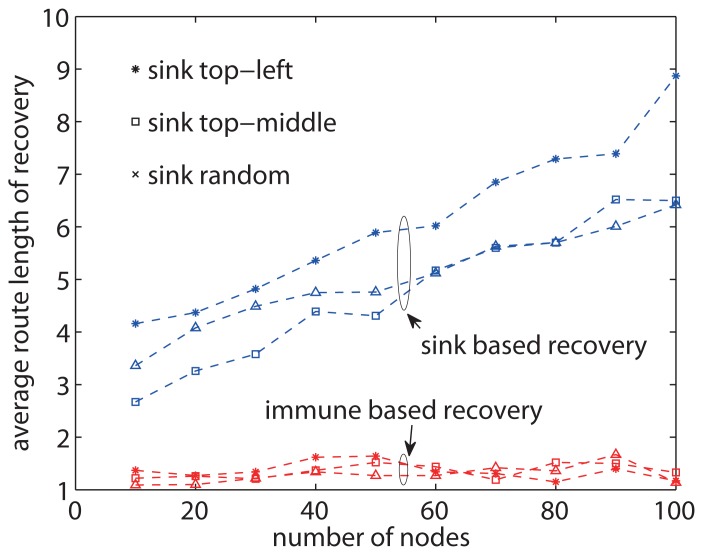
The route length of loss recoveries when the sink node is in the top-left corner, on the top-middle edge, or at random position.

Figure 10 shows the results of recovery route length with predefined positions of the sink node. The route length to sink-based recovery increases as the number of nodes increases. On the other hand, the route length of the proposed approach shows hardly any increase even when the number of nodes becomes large. Although the location of the sink node varies, there are only minor differences between the route lengths. The difference is because the sink position affects the length of route between the object and sink. The route length of the proposed approach varies little regardless of the positions of the sink node. The results reveal that the proposed approach achieves good scalability with regard to the network size, and the positions of the sink node have little impact on the performance of recovery.

We further examine the impact of the number of detectors on the successful recoveries in a shared wireless communication media in which each detector contends for media access in a random manner. Contention-based media access has been widely used in wireless networks due to its simplicity, easy setup, and low requirements especially on small sensor nodes [21,22]. Two types of media contention windows are set up, *i.e.*, 15 time units and 3 time units.

**Figure 11.**
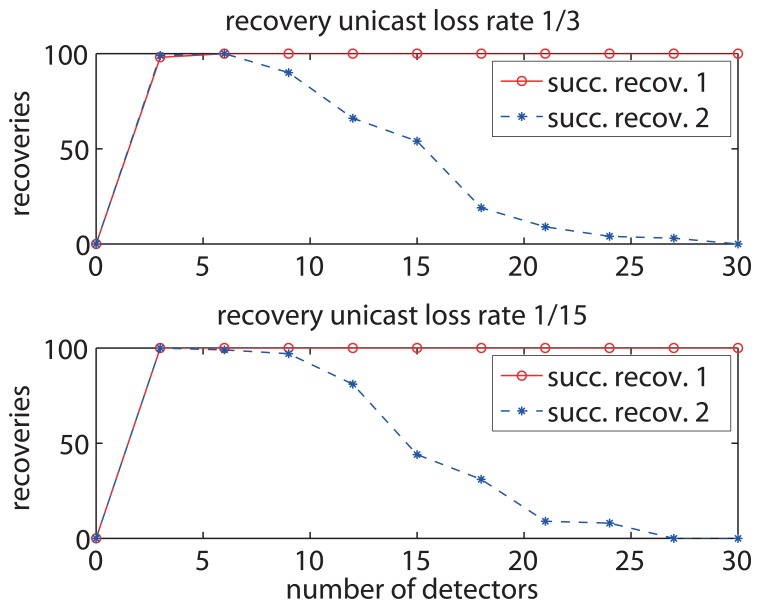
The impact of detector number on loss recoveries with recovery unicast loss rate of 1/15 and 1/3.

As shown in Figure 11, the successful recoveries increase as the number of detectors increases. The result of successful recoveries 1 corresponds to the larger contention window of media access and the result of successful recoveries 2 corresponds to the smaller contention window. The result of successful recoveries 1 reveals that the larger contention window of media access results in and maintains a higher successful recovery rate when the number of detectors is larger than 3. On the other hand, the result of successful recoveries 2 reveals that the network with smaller contention window has a large successful recovery rate only when the detector number is between 3 and 9, and there is a sharp decrease of successful recovery rate when the detector number is larger than 9. This is because the smaller contention window causes more packet conflicts among detectors, leading to packet losses in the recovery operation especially when there are a large number of detectors. Figure 11 also shows the evaluation with the setup of a larger unicast loss rate, which is affected by the basic wireless communication environment such as interference. Larger unicast loss rate results in smaller successful recovery rate in case of small contention window and requires more detectors to get a high successful recovery rate in case of utilizing a large contention window. This is because the larger unicast loss rate leads to more losses of recovery packets.

## 7. Conclusions

Successfully receiving query messages is essential for sensor nodes to collect and report sensing data in response to the queries. Localized discovery and localized recovery of query losses are required for sensor nodes due to the large network size. This paper introduces a localized approach for the discovery and recovery of query losses in sensor networks. We employ the mechanisms of distributed detector clustering, local query name resolution, and collective operation of detectors to achieve both scalability and high success rate of loss discovery and recovery. We proved the scalability and the high successful delivery rate of the proposed approach. We extended the analysis of the approach to the MAC and PHY layers to find appropriate collective operation of loss detectors. Our simulation results imply that the proposed approach achieved short route length of recovery, high successful discovery, and high recovery rates.

The proposed approach can be applied to the on-demand information retrieval in sensor networks. Since it employs a fully distributed and local operation while achieving a global improvement of query dissemination, it has an especially good performance in large size sensor networks and can be applied without depending on specific broadcast mechanisms.
